# Liver abscess caused by Gram-negative spiral bacilli

**DOI:** 10.1099/jmmcr.0.005155

**Published:** 2018-06-08

**Authors:** Hideharu Hagiya, Keigo Kimura, Isao Nishi, Kazunori Tomono

**Affiliations:** ^1^​ Division of Infection Control and Prevention, Osaka University Hospital, 2-15 Yamadaoka, Suita, Osaka 565-0871, Japan; ^2^​ Laboratory for Clinical Investigation, Osaka University Hospital, 2-15 Yamadaoka, Suita, Osaka 565-0871, Japan

**Keywords:** Liver abscess, *Desulfovibrio*

## Case summary

**Fig. 1.**
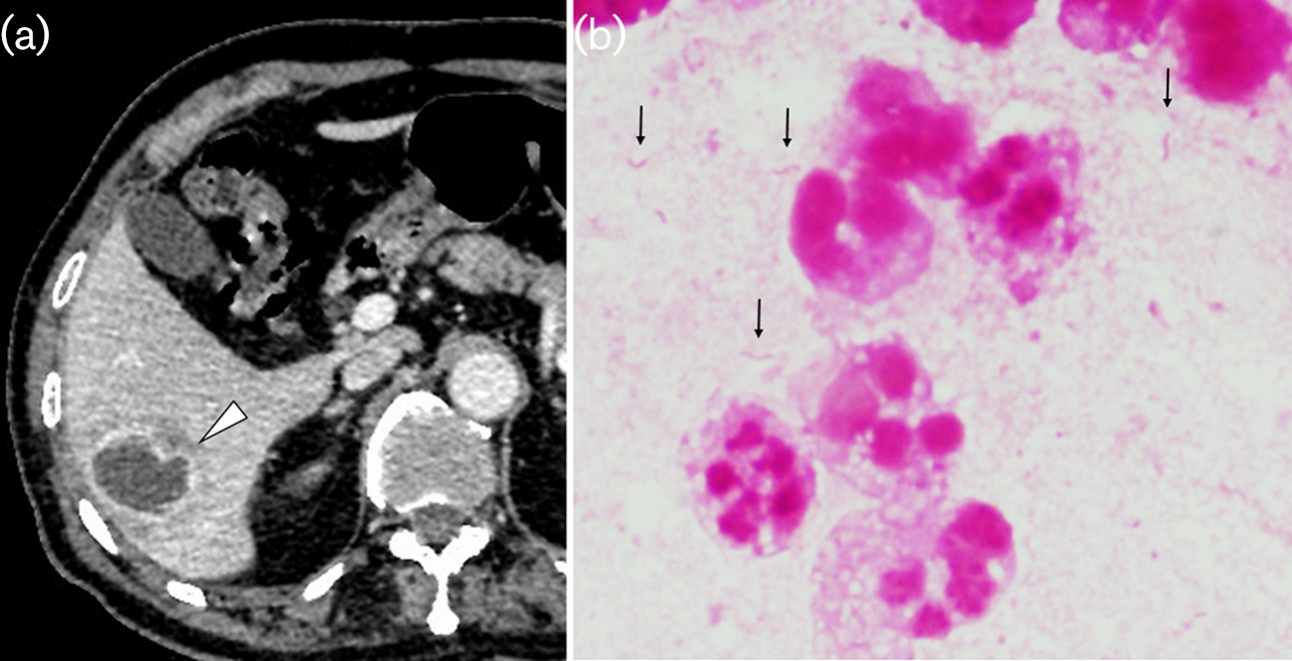
Contrast-enhanced computed tomography showing the liver abscess (a) and Gram-negative spiral bacilli in the pus (b).

A man in his eighties presenting high fever accompanying right flank pain visited us. He had had an episode of several weeks of diarrhoea a month previously. Laboratory analysis showed an elevation of serum C-reactive protein (9.55 mg dl^−1^), and enhanced computed tomography showed a hepatic mass suggesting liver abscess (Fig. 1a). Pus was drained through percutaneous paracentesis, and Gram staining of the purulent material was performed (Fig. 1b).

**Question**

Which of the following organisms is the most plausible pathogen?

**Answer options**

1. *Helicobacter pylori*

2. *Helicobacter cinaedi*

3. *Brachyspira pilosicoli*

4. *Campylobacter fetus*

5. *Desulfovibrio desulfuricans*

## Discussion

**Correct Answer:** 5. *Desulfovibrio desulfuricans.*

The Gram staining shows Gram-negative spiral bacilli. Although species of the genera *Helicobacter* and *Campylobacter* are clinically common Gram-negative spiral bacilli, these pathogens rarely cause liver abscess. The organism was an obligate anaerobe that was positive for the desulfoviridin test and hydrogen sulfide production. The results of 16S rDNA sequence analysis confirmed the organism to be *Desulfovibrio desulfuricans* subsp. *desulfuricans* with concordance rates of 99.7 % (1508/1513 bps) to a reference strain ATCC 27774.

Members of the genus *Desulfovibrio* are anaerobic, Gram-negative, sulfate-reducing bacteria possibly colonizing the human digestive tract [1]. Although its spiral form is characteristic of the organism, identification of species of the genus *Desulfovibrio* is usually difficult due to their rarity and slow growth, leading to under-reporting of the infection [2]. It has been reported that *Desulfovibrio* infections usually involve elderly men with abdominal illnesses, especially hepatobiliary diseases [3]. *Desulfovibrio* infections should be suspected when spiral bacilli are detected in anaerobic culture deriving intra-abdominal samples [4, 5]. The patient recovered well after treatment with cefoperazone/sulbactam, followed by oral metronidazole.
